# Supplementary material for: ACtivE: Assembly and CRISPR-Targeted in Vivo Editing for Yeast Genome Engineering Using Minimum Reagents and Time
Source: ACS Synth Biol. 2022 Oct 17;11(11):3629–43. doi: 10.1021/acssynbio.2c00175 (PMC9680028; doi:10.1021/acssynbio.2c00175)
Supplement: Supplementary file 1 — sb2c00175_si_001.pdf [file sb2c00175_si_001.pdf]

Supporting Information

ACtivE: Assembly and CRISPR-targeted *in vivo* Editing for Yeast Genome Engineering Using Minimum Reagents and Time

Koray Malcı[1,2], Nestor Jonguitud-Borrego[1,2], Hugo van der Straten Waillet[1], Urtė Puodžiūnaitė[1,2,3], Emily J. Johnston[2,3], Susan J. Rosser[2,3], Leonardo Rios-Solis[1,2,4]*

[1] Institute for Bioengineering, School of Engineering, University of Edinburgh, Edinburgh EH9 3BF, UK

[2] Centre for Synthetic and Systems Biology (SynthSys), University of Edinburgh, Edinburgh EH9 3BD, UK

[3] School of Biological Sciences, University of Edinburgh, Edinburgh EH9 3FF, UK

[4] School of Natural and Environmental Sciences, Newcastle University, Newcastle upon Tyne NE1 7RU, UK

* Corresponding author: Dr. Leonardo Rios Solis (email: leo.rios@newcastle.ac.uk)

Table S1: The primer list for *in vivo* CRISPR plasmid assembly and for controlling correct assembly

Name	Sequence 5' to 3'	Purpose
yCas9 + E For	CATACGTTGAAACTACGGCAAAGGATTGGTCAGATCGCTTCATACAGGGAAAGTTCGGCAGAATTCGCATCTAGACTGAACTG	yCas9 amplification with synthetic overlapping sequences
yCas9 + A Rev	GTGCCTATTGATGATCTGGCGGAATGTCTGCCGTGCCATAGCCATGCCTTCACATATAGTGAGGTAGGGCATATGTCCTCTG	
yCas9 + E (ultramer) For	TGTCATACAGCTCAGGGATTGGTCAAGGATTCTTCATACACATACGTTGAAACTACGGCAAAGGATTGGTCAGATCGCTTCATACAGGGAAAGTTCGGCAGAATTCGCATCTAGACTGAACTG	yCas9 amplification with synthetic overlapping sequences and extra bases
yCas9 + A (ultramer) Rev	AGCCATAGCGAATGTCTGTCTGCATTATAGGTCTGCCGTGGTGCCTATTGATGATCTGGCGGAATGTCTGCCGTGCCATAGCCATGCCTTCACATATAGTGAGGTAGGGCATATGTCCTCTG	
yCas9 Internal For	GACATTGTCCTGACTCTCACTCTGTTCGAGGACCGGGAAATGATCGAGGAG	Amplification of half-length yCas9
yCas9 Internal Rev	CTTAAGCCTCTCCTCGATCATTTCCCGGTCCTCGAACAGAGTGAGAGTCAG	
URA3 + A For	ACTATATGTGAAGGCATGGCTATGGCACGGCAGACATTCCGCCAGATCATCAATAGGCACGCAGATTGTACTGAGAGTGCACC	URA3 amplification with synthetic overlapping sequences
URA3 + B Rev	GTTGAACATTCTTAGGCTGGTCGAATCATTTAGACACGGGCATCGTCCTCTCGAAAGGTGCGCATCTGTGCGGTATTTCAC	
E.coli Part + B For	CACCTTTCGAGAGGACGATGCCCGTGTCTAAATGATTCGACCAGCCTAAGAATGTTCAACGTGCGCGGAACCCCTATTTG	Amplification of bacterial fragment with synthetic

E.coli Part + C Rev	CTAGCGTGTCCTCGCATAGTTCTTAGATTGTCGCTACGGCATATACGATCCGTGAGACGTGAGCGGTATCAGCTCACTCAAAG	overlapping sequences
gRNA cassette + C For	ACGTCTCACGGATCGTATATGCCGTAGCGACAATCTAAGAACTATGCGAGGACACGCTAGCCCTCACTAAAGGGAACAAAAGC	Amplification of gRNA cassette with synthetic overlapping sequences
gRNA cassette + D Rev	AATCACTCTCCATACAGGGTTTCATACATTTCTCCACGGGACCCACAGTCGTAGATGCGTGGAAACAACAAAAGGATGTCGAC	
gRNA cassette For	ACGTCTCACGGATCGTATATG	Amplification of gRNA cassette for tandem gRNAs for multiplexing
gRNA cassette + F Rev	AAGGGCCATGACCACCTGATGCACCAATTAGGTAGGTCTGGCTATGTCTATACCTCTGGCTAAATTGGCCATAGAAAAATTCTGTTATC	
gRNA cassette + F For	GCCAGAGGTATAGACATAGCCAGACCTACCTAATTGGTGCATCAGGTGGTCATGGCCCTTTGGAGCTCTTTGAAAAGATAATGTATG	Amplification of gRNA cassette for tandem gRNAs for multiplexing
gRNA cassette + G Rev	GTCACGGGTTCTCAGCAATTCGAGCTATTACCGATGATGGCTGAGGCGTTAGAGTAATCTTAAATTGGCCATAGAAAAATTCTGTTATC	
gRNA cassette + G For	AGATTACTCTAACGCCTCAGCCATCATCGGTAATAGCTCGAATTGCTGAGAACCCGTGACTGGAGCTCTTTGAAAAGATAATGTATG	Amplification of gRNA cassette for tandem gRNAs for multiplexing
gRNA cassette Rev	CAATCACTCTCCATACAGGGTTTC	
2µ ori + D For	ACGCATCTACGACTGTGGGTCCCGTGGAGAAATGTATGAAACCCTGTATGGAGAGTGATTGACGAAAGGGCCTCGTGATAC	Amplification of 2µ ori with synthetic overlapping sequences
2µ ori + E Rev	TGCCGAACTTTCCCTGTATGAAGCGATCTGACCAATCCTTTGCCGTAGTTTCAACGTATGCATTTCCCCGAAAAGTGCCACC	
F1 (A For)	GGAAGTCAAGAAGGACCTTATC	Colony PCR for fragment A
R1 (A Rev)	GTGTGCATTCGTAATGTCTG	
F2 (B For)	CGGCAGAAGAAGTAACAAAG	Colony PCR for fragment B
R2 (B Rev)	GTCATGCCATCCGTAAGATG	
F3 (C For)	CGAACTGAGATACCTACAGC	Colony PCR for fragment C
R3 (C Rev)	CAAGTTGATAACGGACTAGCC	
F4 (D For)	CTATTGTTATGTAAAATGCCACCT	Colony PCR for fragment D
R4 (D Rev)	CACATACAGCTCACTGTTCA	
F5 (E For)	GAAGCACAGATTCTTCGTTGG	Colony PCR for fragment E
R5 (E Rev)	GTTCCTCACTCTTTCCTTACTCA	
Seq1	CCGATAATTGCAGACGAACG	Primer walking for Sanger sequencing
Seq2	CACGAGAAATACCCAACCATC	
Seq3	CCAATGAGAAGGTCCTGCC	
Seq4	GACAATAAGGTGCTGACTCG	
Seq5	CAGAAGGGTAATGAGCTGGC	
Seq6	GCTGCCAAGCTATTTAATATCATGC	
Seq7	CCCAGAAACGCTGGTGAAAG	
Seq8	CCTACATACCTCGCTCTGC	
Seq9	GGAACGCAAACTTCTGTCTAGTGG	
Seq10	CGCAGCTTCGCTAGTAATC	

* yCas9 stands for yeast codon-optimized Cas9 gene
** Black nucleotides represent the annealing part; red nucleotides represent the overlapping part of the primers. Blue nucleotides in the ultramers were used to lengthen the primers, and they do not have any other function.
*** A, B, C, D, and E stand for 60 bp synthetic overlapping fragments

Table S2: The connectors (60 bp synthetic fragments) used for *in vivo* plasmid assembly

Name	Sequence 5' to 3'
**A**	ACTATATGTGAAGGCATGGCTATGGCACGGCAGACATTCCGCCAGATCATCAATAGGCAC
**B**	CACCTTTCGAGAGGACGATGCCCGTGTCTAAATGATTCGACCAGCCTAAGAATGTTCAAC
**C**	ACGTCTCACGGATCGTATATGCCGTAGCGACAATCTAAGAACTATGCGAGGACACGCTAG
**D**	ACGCATCTACGACTGTGGGTCCCGTGGAGAAATGTATGAAACCCTGTATGGAGAGTGATT
**E**	CATACGTTGAAACTACGGCAAAGGATTGGTCAGATCGCTTCATACAGGGAAAGTTCGGCA
**F**	GCCAGAGGTATAGACATAGCCAGACCTACCTAATTGGTGCATCAGGTGGTCATGGCCCTT
**G**	AGATTACTCTAACGCCTCAGCCATCATCGGTAATAGCTCGAATTGCTGAGAACCCGTGAC

* The fragments are orthogonal with 46% < GC < 51% [1]

Table S3: The primer list for *mNeonGreen* integration into eight genomic loci and *mCherry* integration into ARS 1531 region

Name	Sequence 5' to 3'	Purpose
209 UHA For	GCAAAGAGCAATGGCAACAG	Amplification of 209 UHA with an overlapping sequence for *TDH3p*
209 UHA Rev	TATTCTTTGAAATGGCAGTATTGATAATGAC TAGCACATTTTATGGGCCTAAG	
209 *TDH3p* For	ATGATGTCTTAGGCCCATAAAATGTGCTAG TCATTATCAATACTGCCATTTCAAAG	Amplification of promoter *TDH3p* with overlapping sequences for 209 UHA and *mNeonGreen*
TDH3p Rev (**common for all regions**)	CATGTTATCCTCCTCGCCCTTGCTCACCAT TTTGTTTGTTTATGTGTGTTTATTCGAAAC	
mNeonGreen For (**common for all regions**)	GTTTCGAATAAACACACATAAACAAACAAA ATGGTGAGCAAGGGCGAGGA	Amplification of *mNeonGreen* with overlapping sequences for *TDH3p* and 209 DHA
209 *mNeonGreen* Rev	TTGAATACAGAGCAAAAGGATTAGCCATAC CGTTCAGGGTAATATATTTTAACCGCCG	
209 DHA For	GTCGGCGGTTAAAATATATTACCCTGAACG GTATGGCTAATCCTTTTGCTCTG	Amplification of 209 DHA with an overlapping sequence for *mNeonGreen*
209 DHA Rev	CTCTATATCGCTGTTGCTTATGG	
306 UHA For	GTGACTGTCTCCAAGAATACGAC	Amplification of 306 UHA with an overlapping sequence for *TDH3p*
306 UHA Rev	TATTCTTTGAAATGGCAGTATTGATAATGAC GTTATTGATGTTAGGAGAAGGAGC	
306 *TDH3p* For	CTGTTCGTTATTGATGTTAGGAGAAGGAGC TCATTATCAATACTGCCATTTCAAAG	Amplification of promoter *TDH3p* with overlapping sequences for 306 UHA and *mNeonGreen*
306 *mNeonGreen* Rev	TTCAGAAACACTGCTTACACTATTCACCAG CGTTCAGGGTAATATATTTTAACCGCCG	Amplification of *mNeonGreen* with overlapping sequences for *TDH3p* and 306 DHA

306 DHA For	GTCGGCGGTTAAAATATATTACCCTGAACG<br>CTGGTGAATAGTGTAAGCAGTGTTTC	Amplification of 306 DHA with an overlapping sequence for *mNeonGreen*
306 DHA Rev	CAAGAACACCAGACCTCCAAGC	
727 UHA For	CTGCCCCAGGACTTGGAAGG	Amplification of 727 UHA with an overlapping sequence for *TDH3p*
727 UHA Rev	TATTCTTTGAAATGGCAGTATTGATAATGAC<br>ATAGCAGTGGCGCGGTC	
727 *TDH3p* For	TTATAGGGAATCGACCGCGCCACTGCTAT<br>GTCATTATCAATACTGCCATTTCAAAG	Amplification of promoter *TDH3p* with overlapping sequences for 727 UHA and *mNeonGreen*
727 *mNeonGreen* Rev	ATCAGCAGGCCATGGATAAACTTTCCGTTG<br>CGTTCAGGGTAATATATTTTAACCGCCG	Amplification of *mNeonGreen* with overlapping sequences for *TDH3p* and 727 DHA
727 DHA For	GTCGGCGGTTAAAATATATTACCCTGAACG<br>CAACGGAAAGTTTATCCATGG	Amplification of 727 DHA with an overlapping sequence for *mNeonGreen*
727 DHA Rev	GAGATTCTTGACGTAAAGTGC	
1011 UHA For	GTGGTACAAGAAGCGTTGGAGAC	Amplification of 1011 UHA with an overlapping sequence for *TDH3p*
1011 UHA Rev	TATTCTTTGAAATGGCAGTATTGATAATGAC<br>TTCCAGCAGCGCCAGTAG	
1011 *TDH3p* For	GCTCAACAACCCTACTGGCGCTGCTGGAA<br>GTCATTATCAATACTGCCATTTCAAAG	Amplification of promoter *TDH3p* with overlapping sequences for 1011 UHA and *mNeonGreen*
1011 *mNeonGreen* Rev	CCAACACTTGATAGTATCTACTCGCCATTC<br>CGTTCAGGGTAATATATTTTAACCGCCG	Amplification of *mNeonGreen* with overlapping sequences for *TDH3p* and 1011 DHA
1011 DHA For	GTCGGCGGTTAAAATATATTACCCTGAACG<br>GAATGGCGAGTAGATACTATCAAG	Amplification of 1011 DHA with an overlapping sequence for *mNeonGreen*
1011 DHA Rev	CTTCACATTGAGTTTGAATATGCC	
1316 UHA For	GGTTTCAAGCCAAATTGTACG	Amplification of 1316 UHA with an overlapping sequence for *TDH3p*
1316 UHA Rev	TATTCTTTGAAATGGCAGTATTGATAATGAC<br>TTAGGTAGTAACTATACGCAGC	
1316 *TDH3p* For	GGAGCAGCTGCGTATAGTTACTACCTAAGT<br>CATTATCAATACTGCCATTTCAAAG	Amplification of promoter *TDH3p* with overlapping sequences for 1316 UHA and *mNeonGreen*
1316 *mNeonGreen* Rev	TAGCCCACTTCTAGCCAACTTCTAGCCCAC<br>CGTTCAGGGTAATATATTTTAACCGCCG	Amplification of *mNeonGreen* with overlapping sequences for *TDH3p* and 1316 DHA
1316 DHA For	GTCGGCGGTTAAAATATATTACCCTGAACG<br>GTGGGCTAGAAGTTGGCTAGAAG	Amplification of 1316 DHA with an overlapping sequence for *mNeonGreen*
1316 DHA Rev	GCGCATAGTGCTAGTCTTTTCTCC	
1406 UHA For	GTTGGTATTCTCGATAGGCAGC	Amplification of 1406 UHA with an overlapping sequence for *TDH3p*
1406 UHA Rev	TATTCTTTGAAATGGCAGTATTGATAATGAC<br>CCATCAGAACCGTAAACCTTG	
1406 *TDH3p* For	GAAAGCGCCAAGGTTTACGGTTCTGATGG<br>GTCATTATCAATACTGCCATTTCAAAG	Amplification of promoter *TDH3p* with overlapping sequences for 1406 UHA and *mNeonGreen*
1406 *mNeonGreen* Rev	AAGGATACTTCAAGACTAGATTCCCCCCTG<br>CGTTCAGGGTAATATATTTTAACCGCCG	Amplification of *mNeonGreen* with overlapping sequences for *TDH3p* and 1406 DHA
1406 DHA For	GTCGGCGGTTAAAATATATTACCCTGAACG<br>CAGGGGGGAATCTAGTCTTG	Amplification of 1406 DHA with an overlapping sequence for *mNeonGreen*
1406 DHA Rev	GCGTCCTTATCGAAAGGAAC	
1531 UHA For	GACTGCCTCTTGATGTTATGCCA	Amplification of 1531 UHA with an overlapping sequence for *TDH3p*
1531 UHA Rev	TATTCTTTGAAATGGCAGTATTGATAATGAG<br>AAAGTTGCCGAGGCCAAATG	

1531 *TDH3p* For	TATTTTCTCCATTTGGCCTCGGCAACTTTCTCATTATCAATACTGCCATTTCAAAG	Amplification of promoter *TDH3p* with overlapping sequences for 1531 UHA and *mNeonGreen*
1531 *mNeonGreen* Rev	ACGTAGATCGGTATATACGTTCAAGCCCCCCGTTCAGGGTAATATATTTTAACCGCCG	Amplification of *mNeonGreen* with overlapping sequences for *TDH3p* and 1531 DHA
1531 DHA For	GTCGGCGGTTAAAATATATTACCCTGAACGGGGGGCTTGAACGTATATACC	Amplification of 1531 DHA with an overlapping sequence for *mNeonGreen*
1531 DHA Rev	GGATGGCAGAACCGATACTAATG	
1603 UHA For	GGCTATGGTGGTGATGTCTG	Amplification of 1603 UHA with an overlapping sequence for *TDH3p*
1603 UHA Rev	TATTCTTTGAAATGGCAGTATTGATAATGAGAGGAAAAAAAAACAGTTGTACATTGG	
1603 *TDH3p* For	GTTACCAATGTACAACTGTTTTTTTTTCCTCTCATTATCAATACTGCCATTTCAAAG	Amplification of promoter *TDH3p* with overlapping sequences for 1603 UHA and *mNeonGreen*
1603 *mNeonGreen* Rev	AAAAAGCTCGTGAATACAGCAAGAACGAAGCGTTCAGGGTAATATATTTTAACCGCCG	Amplification of *mNeonGreen* with overlapping sequences for *TDH3p* and 1603 DHA
1603 DHA For	GTCGGCGGTTAAAATATATTACCCTGAACGCTTCGTTCTTGCTGTATTCACG	Amplification of 1603 DHA with an overlapping sequence for *mNeonGreen*
1603 DHA Rev	CTGTCTCCGCTATGTCAGTTAC	
1531 UHA For	GACTGCCTCTTGATGTTATGCCA	Amplification of 1531 UHA with an overlapping sequence for *TDH3p*
1531 UHA Rev	TATTCTTTGAAATGGCAGTATTGATAATGAGAAAGTTGCCGAGGCCAAATG	
1531 *TDH3p* For	TATTTTCTCCATTTGGCCTCGGCAACTTTCTCATTATCAATACTGCCATTTCAAAG	Amplification of promoter *TDH3p* with overlapping sequences for 1531 UHA and *mCherry*
TDH3p (*mCherry*) Rev	CATGTTATCCTCCTCGCCCTTGCTCACCATTTTGTTTGTTTATGTGTGTTTATTCGAAAC	
1531 *mCherry* For	GTTTCGAATAAACACACATAAACAAACAAAATGGTGAGCAAGGGCGAGG	Amplification of *mCherry* with overlapping sequences for *TDH3p* and 1603 DHA
1531 *mCherry* Rev	ACGTAGATCGGTATATACGTTCAAGCCCCCGGCTGGGAAGCATATTTGAGAAG	
1531 DHA For	GCCGCATCTTCTCAAATATGCTTCCCAGCCGGGGGCTTGAACGTATATACC	Amplification of 1531 DHA with an overlapping sequence for *mCherry*
1531 DHA Rev	GGATGGCAGAACCGATACTAATG	

* Black nucleotides represent the annealing part; red nucleotides represent the overlapping part of the primers.
** *TDH3p* Rev and *mNeonGreen* For primers were the same for all regions

Table S4: The primer list for multiple integrations of the β–carotene pathway genes and for colony PCR to control integrations

Name	Sequence 5' to 3'	Purpose
1406 UHA For	GTTGGTATTCTCGATAGGCAGC	Amplification of 1406 UHA with an overlapping sequence for *TDH3p*
1406 UHA Rev	TATTCTTTGAAATGGCAGTATTGATAATGACCCATCAGAACCGTAAACCTTG	
1406 *TDH3p* For	GAAAGCGCCAAGGTTTACGGTTCTGATGGGTCATTATCAATACTGCCATTTCAAAG	Amplification of promoter *TDH3p* with overlapping sequences for 1406 UHA and *CrtE*
TDH3p (*CrtE*) Rev	GATAGCGGTCAAGATGTTAGCGTAGTCCATTTTGTTTGTTTATGTGTGTTTATTCGAAAC	
CrtE For	GTTTCGAATAAACACACATAAACAAACAAAATGGACTACGCTAACATCTTGACCG	Amplification of *CrtE* with overlapping sequences for *TDH3p and* 1406 DHA
CrtE Rev	AAGGATACTTCAAGACTAGATTCCCCCCTGCGTTCAGGGTAATATATTTTAACCGCCG	
1406 DHA For	GTCGGCGGTTAAAATATATTACCCTGAACGCAGGGGGGAATCTAGTCTTG	Amplification of 1406 DHA with an overlapping sequence for *CrtE*
1406 DHA Rev	GCGTCCTTATCGAAAGGAAC	
1531 UHA For	GACTGCCTCTTGATGTTATGCCA	Amplification of 1531 UHA with an overlapping sequence for *TDH3p*
1531 UHA Rev	TATTCTTTGAAATGGCAGTATTGATAATGAGAAAGTTGCCGAGGCCAAATG	
1531 *TDH3p* For	TATTTTCTCCATTTGGCCTCGGCAACTTTCTCATTATCAATACTGCCATTTCAAAG	Amplification of promoter *TDH3p* with overlapping sequences for 1531 UHA and *CrtYB*
TDH3p (*CrtYB*) Rev	GTGGATTTGGTAGTAAGCCAAAGCGGTCATTTTGTTTGTTTATGTGTGTTTATTCGAAAC	
CrtYB For	GTTTCGAATAAACACACATAAACAAACAAAATGACCGCTTTGGCTTACTAC	Amplification of *CrtYB* with overlapping sequences for *TDH3p and* 1531 DHA
CrtYB Rev	ACGTAGATCGGTATATACGTTCAAGCCCCCCGTTCAGGGTAATATATTTTAACCGCCG	
1531 DHA For	GTCGGCGGTTAAAATATATTACCCTGAACGGGGGGCTTGAACGTATATACC	Amplification of 1531 DHA with an overlapping sequence for *CrtYB*
1531 DHA Rev	GGATGGCAGAACCGATACTAATG	
1603 UHA For	GGCTATGGTGGTGATGTCTG	Amplification of 1603 UHA with an overlapping sequence for *TDH3p*
1603 UHA Rev	TATTCTTTGAAATGGCAGTATTGATAATGAGAGGAAAAAAAACAGTTGTACATTGG	
1603 *TDH3p* For	GTTACCAATGTACAACTGTTTTTTTTCCTCTCATTATCAATACTGCCATTTCAAAG	Amplification of promoter *TDH3p* with overlapping sequences for 1603 UHA and *CrtI*
TDH3p (*CrtI*) Rev	TGGCTTGTCTTGGTCTTGTTCCTTACCCATTTTGTTTGTTTATGTGTGTTTATTCGAAAC	
CrtI For	GTTTCGAATAAACACACATAAACAAACAAAATGGGTAAGGAACAAGACCAAG	Amplification of *CrtI* with overlapping sequences for *TDH3p and* 1603 DHA
CrtI Rev	AAAAAGCTCGTGAATACAGCAAGAACGAAGCGTTCAGGGTAATATATTTTAACCGCCG	
1603 DHA For	GTCGGCGGTTAAAATATATTACCCTGAACGCTTCGTTCTTGCTGTATTCACG	Amplification of 1603 DHA with an overlapping sequence for *CrtI*
1603 DHA Rev	CTGTCTCCGCTATGTCAGTTAC	
CrtE CDS Rev	CCTCATCAAGATTGCTTTATGCCACTCGAGctaCAATGGGATGTCAGCCAACTTCTTC	OE PCR primers for assembly of *CrtE* CDS and terminator *TDH1t*
TDH1t (*CrtE*) For	TTGAAGAAGTTGGCTGACATCCCATTGtagCTCGAGTGGCATAAAGCAATCTTG	
CrtYB CDS Rev	CCTCATCAAGATTGCTTTATGCCACTCGAGctaTTGACCTTCCCAACCAGACA	OE PCR primers for assembly of *CrtYB* CDS and terminator *TDH1t*
TDH1t (*CrtYB*) For	GTTGTTATGTCTGGTTGGGAAGGTCAAtagCTCGAGTGGCATAAAGCAATCTTG	
CrtI CDS Rev	CCTCATCAAGATTGCTTTATGCCACTCGAGctaGAAAGCCAAAACACCAACAGATC	OE PCR primers for assembly of *CrtI* CDS and terminator *TDH1t*
TDH1t (*CrtI*) For	GCTCGATCTGTTGGTGTTTTGGCTTTCtagCTCGAGTGGCATAAAGCAATCTTG	

CrtE Col PCR For	GAAGAAGTTGGCTGACATCC	Colony PCR for *CrtE* integration
1406 Col PCR Rev	GCGTCCTTATCGAAAGGAAC	
CrtYB Col PCR For	GTTGTTATGTCTGGTTGGGAAGG	Colony PCR for *CrtE* integration
1531 Col PCR Rev	GGATGGCAGAACCGATACTAATG	
CrtI Col PCR For	GCTCGATCTGTTGGTGTTTTG	Colony PCR for *CrtE* integration
1603 Col PCR Rev	CTGTCTCCGCTATGTCAGTTAC	

* Black nucleotides represent the annealing part; red nucleotides represent the overlapping part of the primers.
** The gene names in the parentheses show that the corresponding primer contains overlapping parts for the gene in the parenthesis.

Table S5: The primer and crRNA list for genomic deletions

Name	Sequence 5' to 3'	Purpose
crRNA *GAL80*	GATGAGCGTGGTAACCGATT ***GGG***	20 bp crRNA for *GAL80* deletion
GAL80 DEL UHA For	GCCTGTCTACAGGATAAAGACG	Amplification of UHA for *GAL80* deletion with an overlapping sequence for DHA
GAL80 DEL UHA Rev	ACTGGGGGCCAAGCACAGGGCAAGATGCTTGA CGGGAGTGGAAAGAACGG	
GAL80 DEL DHA For	AGTTGGTTTCCCGTTCTTTCCACTCCCGTCAAG CATCTTGCCCTGTGCTTG	Amplification of DHA for *GAL80* deletion with an overlapping sequence for UHA
GAL80 DEL DHA Rev	CCAGCAAAAATATGACCCCC	
crRNA *DIT1*	CCTTGTCAAGATATATCCAC ***CGG***	20 bp crRNA for *DIT1* deletion
DIT1 DEL UHA For	GAGTCCCTGGAAGGAAAATTATTG	Amplification of UHA for *DIT1* deletion with an overlapping sequence for DHA
DIT1 DEL UHA Rev	TATTCCCCCTCTGTTAAATGGAATTGTGTGGCG GAGGAGCACAATTTATG	
DIT1 DEL DHA For	TAAATTTTCACATAAATTGTGCTCCTCCGCCACA CAATTCCATTTAACAGAGG	Amplification of DHA for *DIT1* deletion with an overlapping sequence for UHA
DIT1 DEL DHA Rev	CTGATGCCTCAAGATTTAACC	

* Black nucleotides represent the annealing part; red nucleotides represent the overlapping part of the primers.
** The **bold** and *italic* sequences show the PAM sequences of corresponding crRNAs

A)

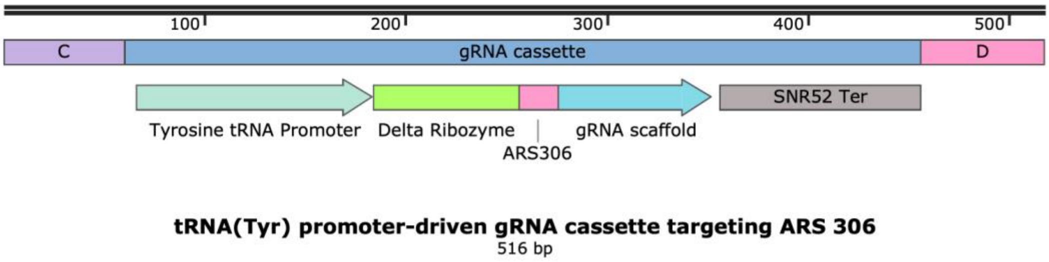

B)

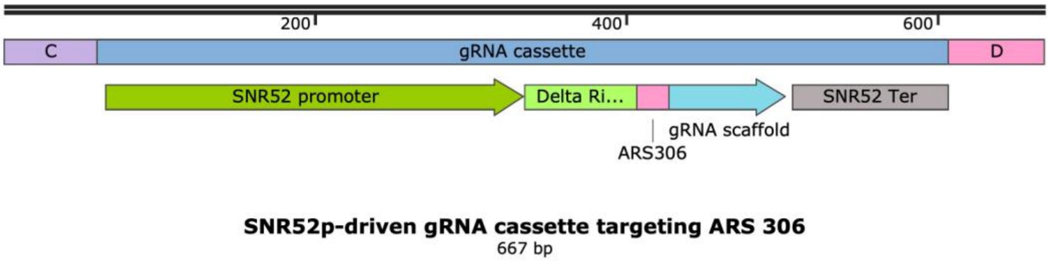

Figure S1: The sequence maps of gRNA cassettes driven by different promoters. **A)** The gRNA cassette is expressed through the $tRNA^{Tyr}$ promoter. It targets ARS 306 on chromosome III. The total length of is 516 bp. **B)** The gRNA cassette is expressed through *SNR52p*. It targets ARS 306 on chromosome III. The total size is 667 bp. C and D represent the 60 bp orthogonal fragments used for *in vivo* DNA assembly with the other plasmid parts. The maps were illustrated using SnapGene®. [2]

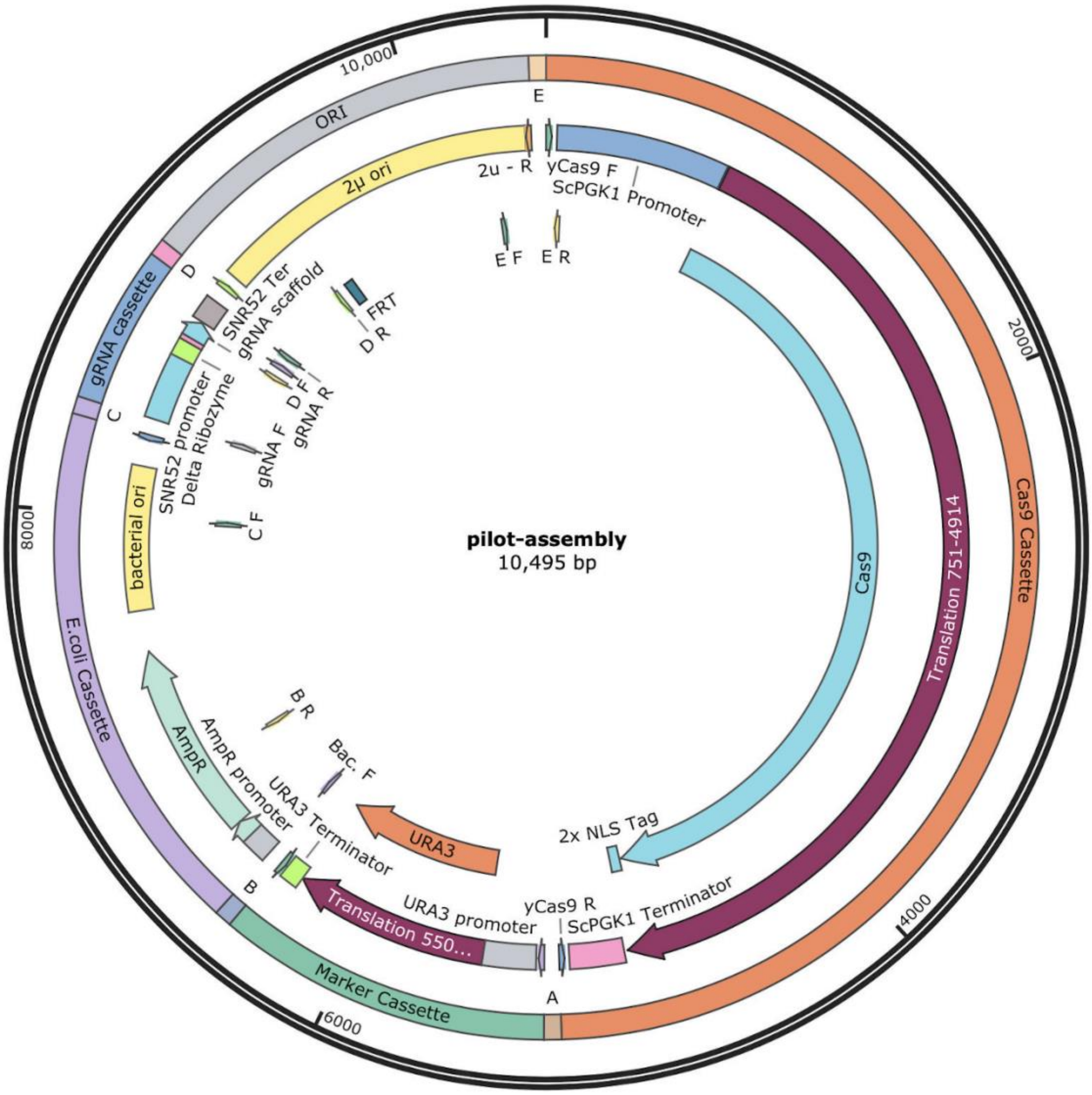

Figure S2: The plasmid map of correct *in vivo* DNA assembly. The short arrows containing F (forward) or R (reverse) suffixes represent primers, while A, B, C, D, and E represent 60 bp synthetic fragments. The primers contain 60 bp synthetic overlapping regions with the adjacent fragment. The system was designed using Benchling.[3] The map was illustrated using SnapGene®. [2]

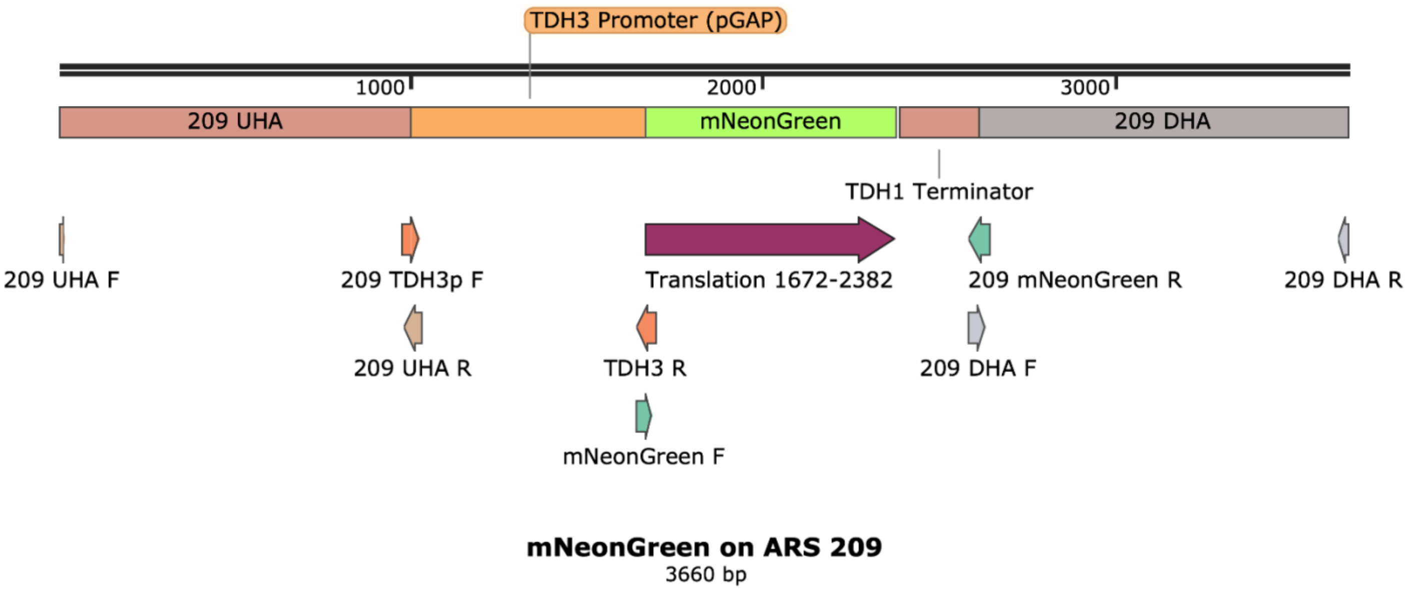

Figure S3: The sequence map of the donor DNA after *in vivo* DNA assembly. ARS 209 region is shown in the figure as an example. A similar design was also used for the other regions. Arrows represent primers. Longer primers contain overlapping regions with the adjacent fragment. The system was designed using Benchling.[3] The map was illustrated using SnapGene®. [2] F: forward primer, R: reverse primer

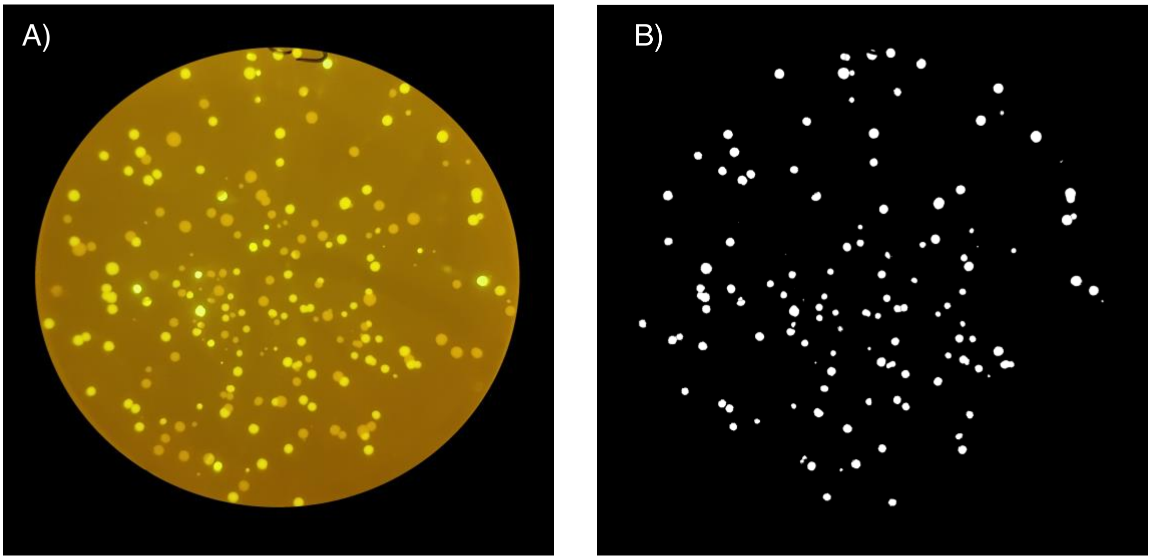

Figure S4: Automated counting of mNeonGreen expressing positive colonies using ImageJ and its Colony Counter plug-in. [4,5] **A)** The first image taken on a blue-LED transilluminator shows both fluorescent mNeonGreen-expressing and non-fluorescent false-positive colonies. **B)** The processed image to automatically select the positive colonies. The image was converted to a 16-bit format, the background and white negative colonies were removed using color threshold, the merged colonies were segmented to obtain individual colonies.

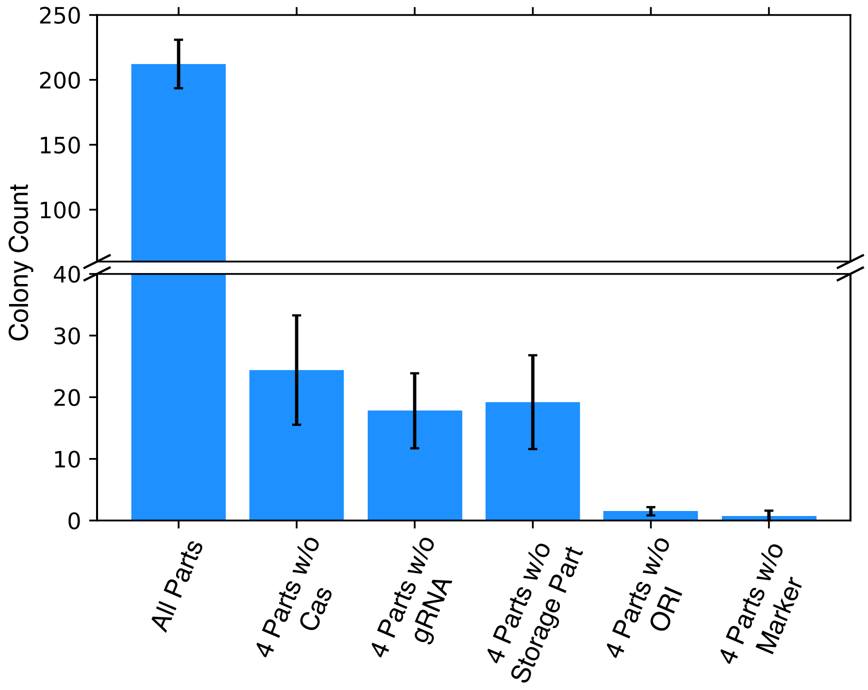

Figure S5: Colony numbers following transformation with different plasmid part combinations. The same transformation conditions were used for each combination. More than 200 colonies were obtained when all parts were used as they had overlapping sequences for *in vivo* assembly via a homology-directed repair mechanism. The error bars represent the standard deviations of three independent replicates.

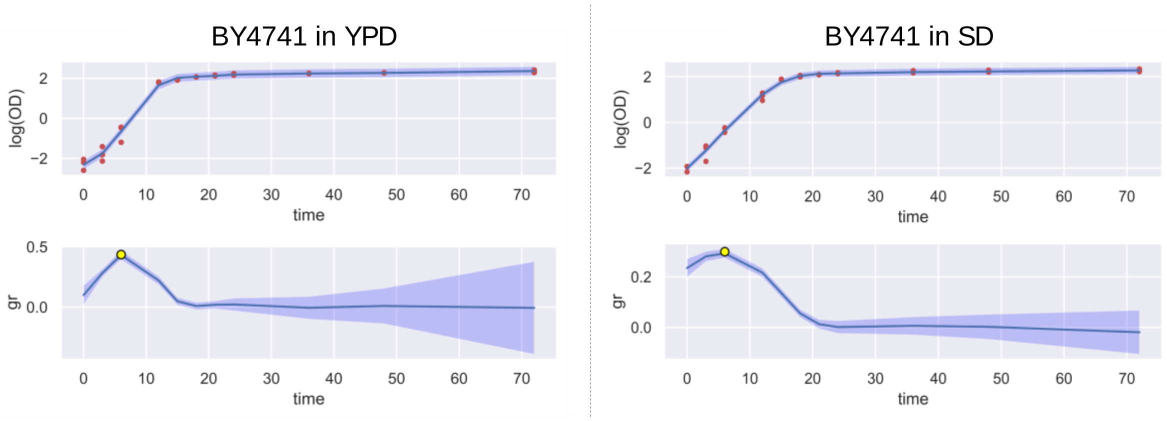

Figure S6: Logarithmic OD_{600} (log(OD)) and the growth rate (gr) of the parental strain, BY4741, in YPD and SD media, respectively, over 72 hours (time). The yellow dots represent the maximum point of the curves. The standard deviations of three independent colonies are shown by shading.

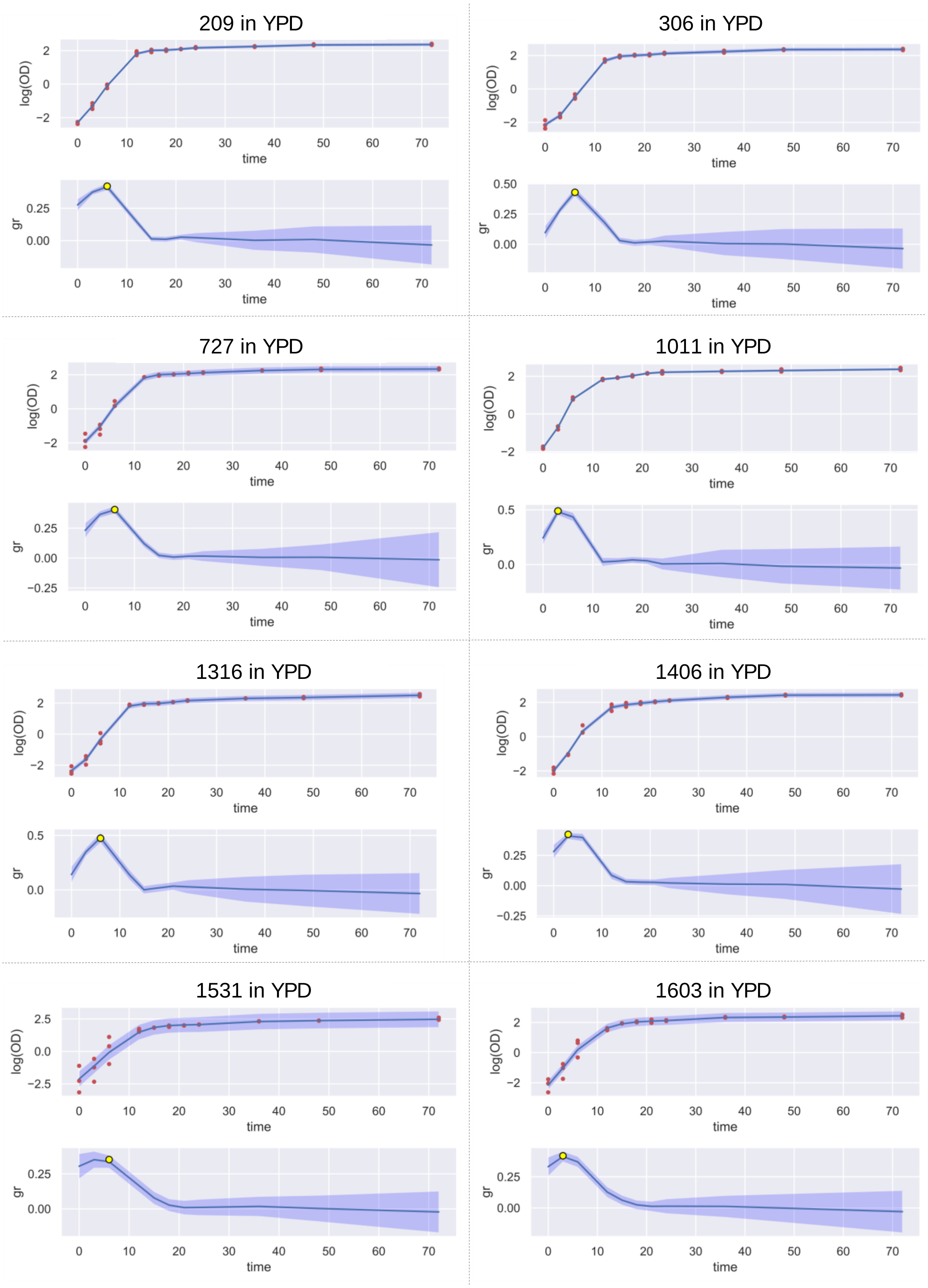

Figure S7: Logarithmic OD_{600} (log(OD)) and growth rates (gr) of *mNeonGreen* integrated strains in YPD media over 72 hours (time). The yellow dots represent the maximum point of the curves. The standard deviations of three independent colonies are shown by shading.

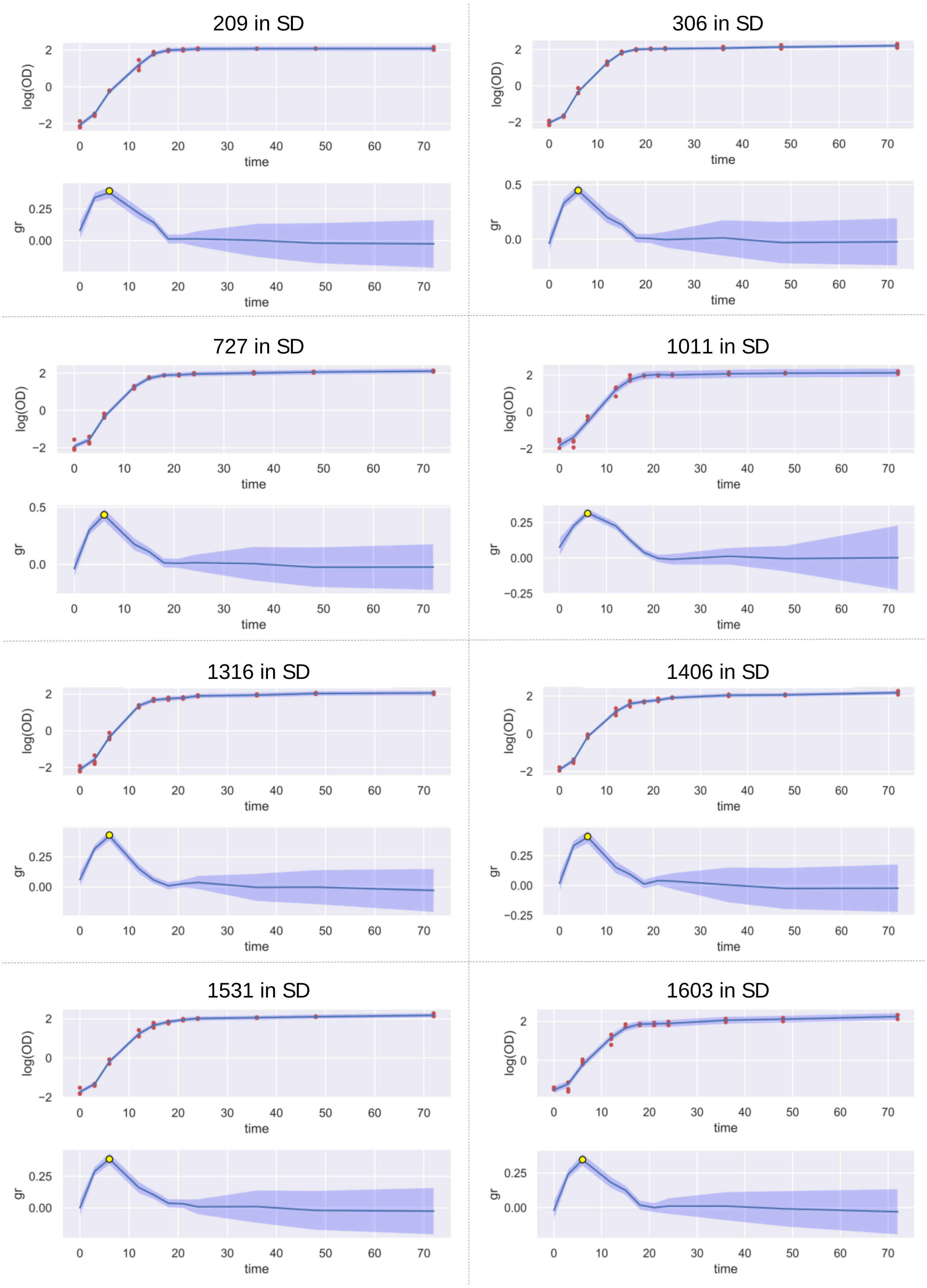

Figure S8: Logarithmic OD_{600} (log(OD)) and growth rates (gr) of *mNeonGreen* integrated strains in SD media over 72 hours (time). The yellow dots represent the maximum point of the curves. The standard deviations of three independent colonies are shown by shading.

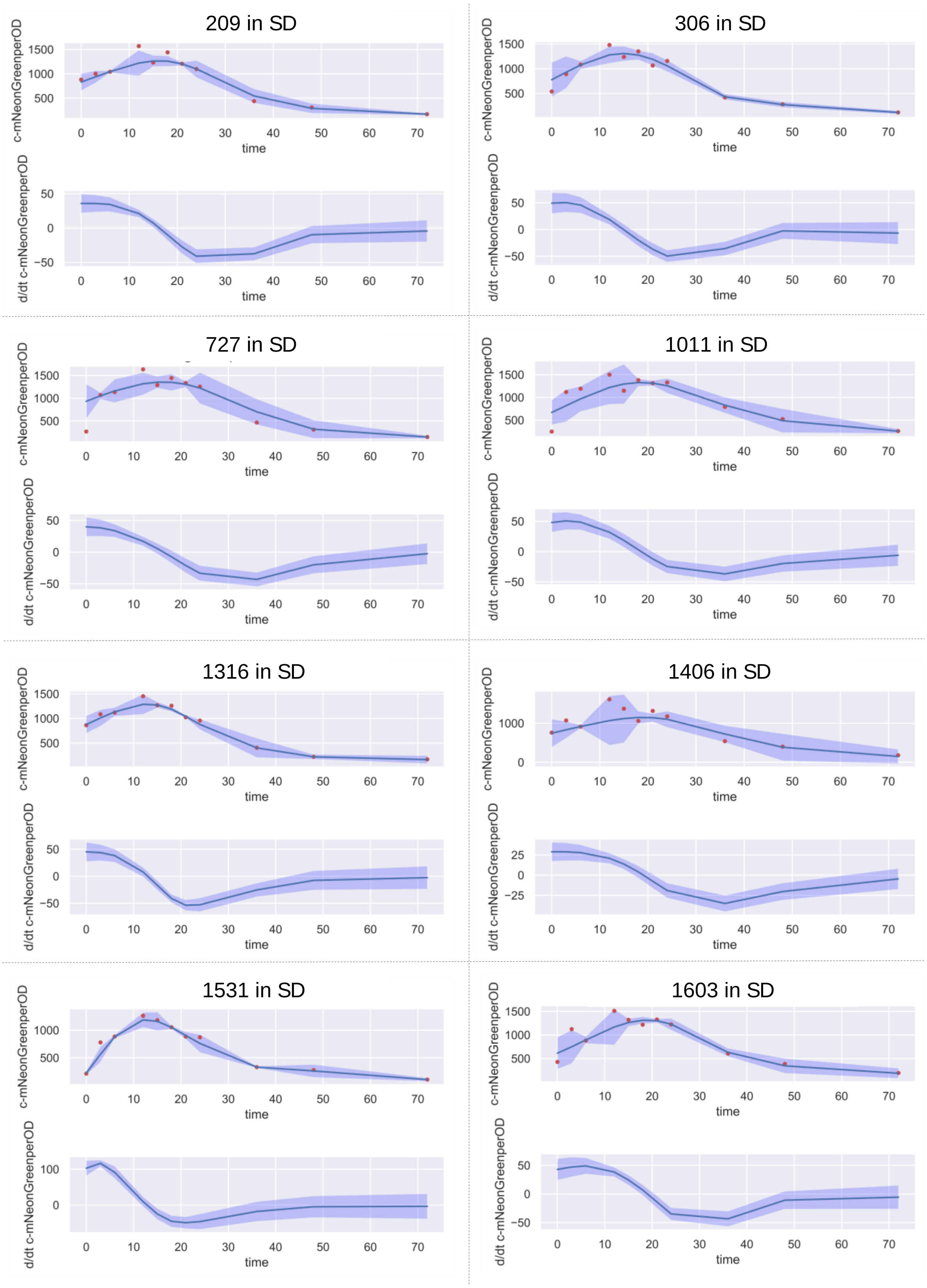

Figure S9: Corrected mNeonGreen expression per OD (c-mNeonGreenperOD) and time-derivative estimations of mNeonGreen expressions (d/dt -mNeonGreenperOD) of *mNeonGreen* integrated strains over 72 hours (time) in SD media. The expressions are shown in the relative fluorescence unit (RFU). The standard deviations of three independent colonies are shown by shading.

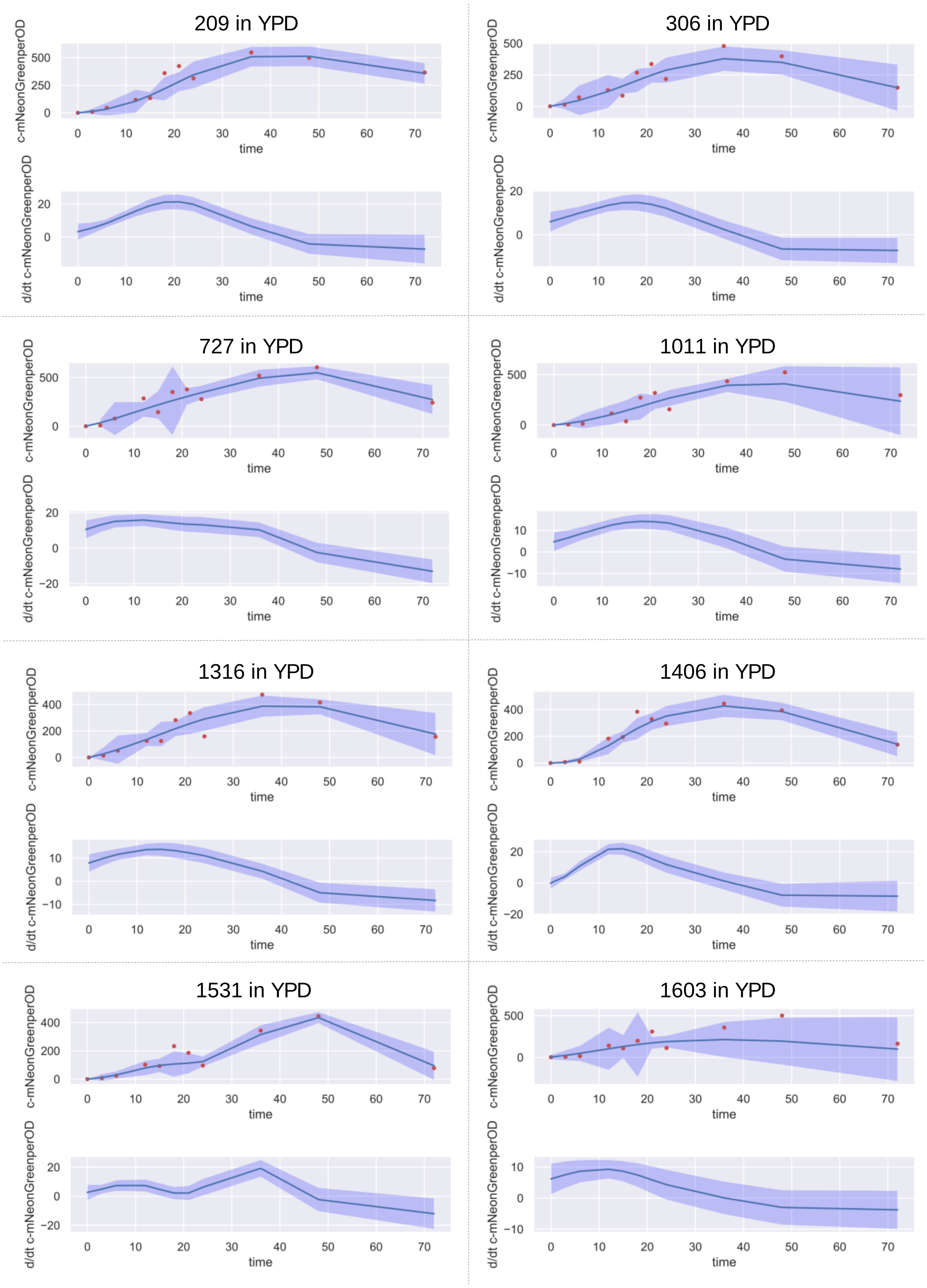

Figure S10: Corrected mNeonGreen expression per OD (c-mNeonGreenperOD) and time-derivative estimations of mNeonGreen expressions (d/dt -mNeonGreenperOD) of *mNeonGreen* integrated strains over 72 hours (time) in YPD media. The expressions are shown in the relative fluorescence unit (RFU). The standard deviations of three independent colonies are shown by shading.

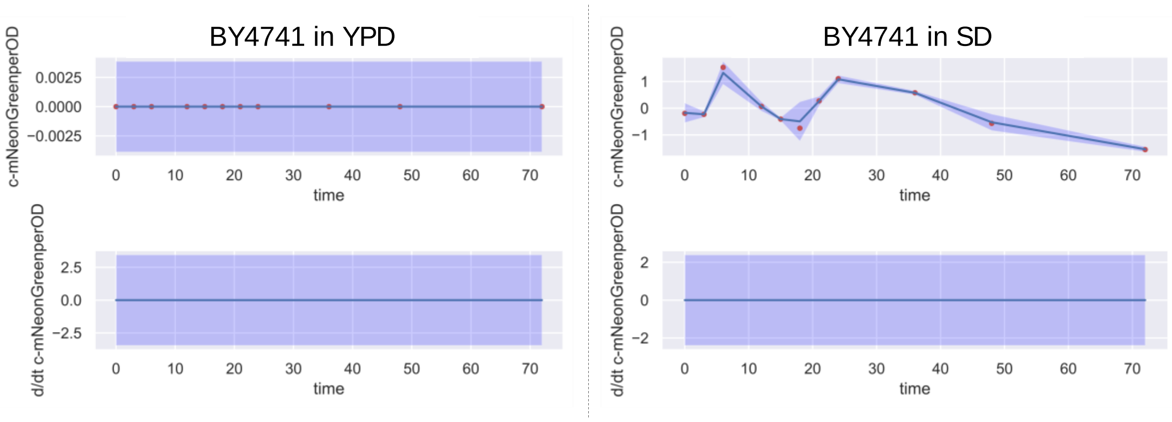

Figure S11: Corrected mNeonGreen expression per OD (c-mNeonGreenperOD) and time-derivative estimations of mNeonGreen expressions (d/dt -mNeonGreenperOD) of parental strain, BY4741, over 72 hours (time). The expressions are shown in the relative fluorescence unit (RFU). The standard deviations of three independent colonies are shown by shading.

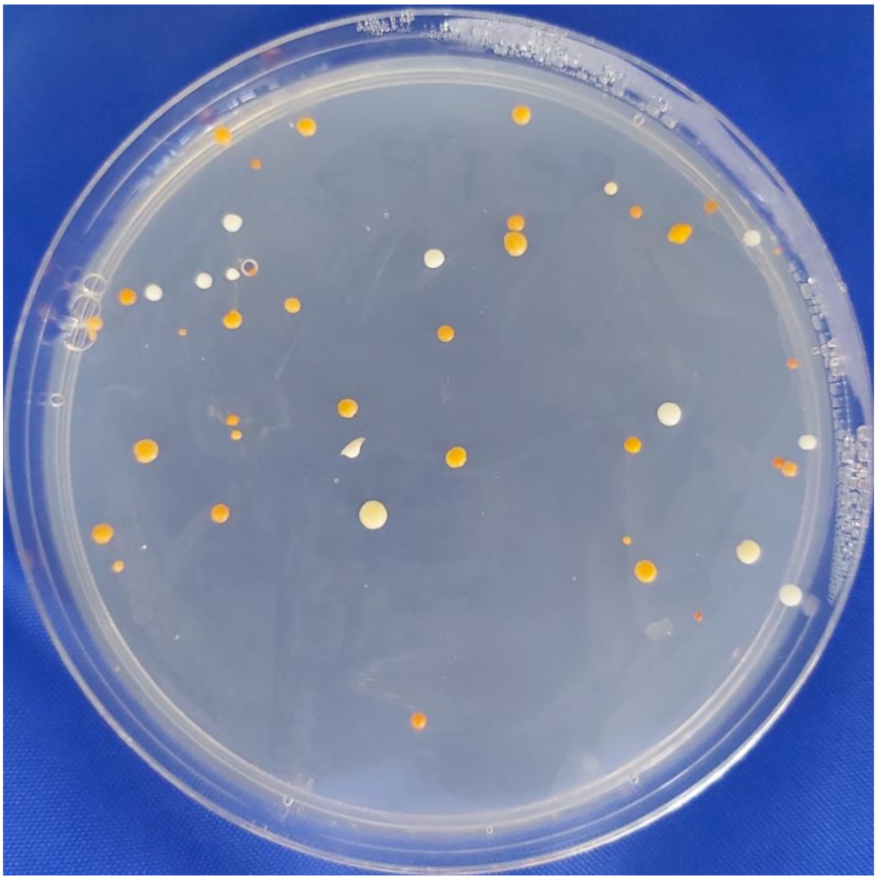

Figure S12: Multi-gene integration of three heterologous genes from the β-carotene pathway onto the ARS1531 region using ACtivE. The orange colonies show the β-carotene production by the correctly integrated genes, whereas the white or yellowish colonies have the missing gene(s) in their genome.

REFERENCES

(1) Kuijpers, N. G.; Solis-Escalante, D.; Bosman, L.; van den Broek, M.; Pronk, J. T.; Daran, J.-M.; Daran-Lapujade, P. A Versatile, Efficient Strategy for Assembly of Multi-Fragment Expression Vectors in Saccharomyces Cerevisiae Using 60 Bp Synthetic Recombination Sequences. *Microb. Cell Factories 2013 121* **2013**, *12* (1), 1–13. https://doi.org/10.1186/1475-2859-12-47.
(2) SnapGene | Software for everyday molecular biology https://www.snapgene.com/ (accessed Aug 28, 2021).
(3) Cloud-Based Informatics Platform for Life Sciences R&D | Benchling https://www.benchling.com/ (accessed Aug 28, 2021).
(4) Colony Counter https://imagej.nih.gov/ij/plugins/colony-counter.html (accessed Aug 29, 2021).
(5) Schneider, C. A.; Rasband, W. S.; Eliceiri, K. W. NIH Image to ImageJ: 25 Years of Image Analysis. *Nat. Methods 2012 97* **2012**, *9* (7), 671–675. https://doi.org/10.1038/nmeth.2089.
